# The impact of COVID-19 on the surgical operations

**DOI:** 10.1016/j.amsu.2020.06.042

**Published:** 2020-07-15

**Authors:** Tagleb S. Mazahreh, Abdelwahab J. Aleshawi, Nabil A. Al-Zoubi, Moad Hatamleh, Alaa Hmedat

**Affiliations:** a Department of General Surgery and Urology, Faculty of Medicine, Jordan Universityof Science & Technology, Irbid, 22110, Jordan; b Intern, King Abdullah University Hospital, Irbid, 22110, Jordan

**Keywords:** Surgery, COVID-19, Jordan, Emergency

In the time span of 4 months, everything has changed in the life. The pandemic of coronavirus disease 2019 (COVID-19) has laid waste to the daily routines we practiced automatically. The most basic assumptions of how we plan our day, and how we practice medicine are gone, and in their place we socially isolate, and we split shifts to decrease exposure. The global impact of this invisible virus is terrifying, and we as physicians and healthcare providers are on the front lines of this war, confronting our own mortality as we continually strategize to protect our patients. We have partnered with our colleagues in infectious disease, respiratory medicine, public health, and administrators to learn quickly, adapt our personal and institutional practice, and adopt policies to allow a new best practice, conserve personal protective equipment (PPE), and save our patients and ourselves. One of the main fields that is affected promptly by this virus is the surgery with its all fields and specialties as most policies within the outbreak recommends decreasing the surgical activity by cease the elective operation and maintain only the emergency surgical cases.

Many authors have shared their experience regarding the surgical practice during COVID-19. One study from Italy investigated the impact of COVID-19 on the surgical practice [[1], [2], [3], [4], [5], [6], [7]]. They report that during the month preceding the quarantine, 82 patients underwent surgical emergency operations: 19 appendectomies, 17 colorectal resections, 17 small bowel surgeries, 11 cholecystectomies, 5 thoracic procedures for spontaneous pneumothorax and strangulated diaphragmatic hernia, 2 gastric resections, and 11 minor procedures [1]. During the month after the quarantine, the emergency surgery volume dropped to the number of 12 cases: 7 appendectomies, one foot amputation, one colostomy, 2 small bowel resections, and one cholecystectomy. They observed a 86% decrease of cases of emergency surgery compared to the month before the quarantine [1].

In Jordan, King Abdullah University Hospital is a tertiary educational center serves the health and care for all northern in Jordan. The quarantine was initiated in Jordan in 18/3/2020. The impact of COVID-19 on the surgical operation is investigated for a period of one month (from 18/3/2020 to 18/4/2020) and compared to surgical record of our center one month before the quarantine. During this month, a total of 183 operations were performed; 121 of them were females. The mean age for the patients 37.1 years; the youngest patient was a 1-day female neonate for myelomeningocele repair and the oldest was an 88-year old female for lower limb embolectomy. 102 of the cases were managed as a mandatory elective case such as the elective cesarean section or oncological cases and 81 were emergency cases. Obstetrics and Gynecology services serve the most frequent operations with 76 case. Also, following operations were performed: 19 for general surgery (including the oncological operations), 16 for neurosurgery, 18 for urology, 16 for vascular surgery, 16 for orthopedics, 7 for ophthalmology, 7 for pediatrics surgery, 3 for maxillo-facial surgery, 2 for thoracic surgery, 2 for otolaryngology and one cardiac surgery. Among the 81 emergency operations, 13 operation were done due to trauma. Cesarean section was the most frequent performed operation with 65 case. Regarding the type of anesthesia, the aim was to utilize the spinal or local anesthesia as much as possible. The spinal and local anesthesia were conducted in 63 and 15 case, respectively. Only one intraoperative complication was reported (bladder injury in cesarean section). Postoperatively, 7 patients developed inpatient complications mostly (6 cases) in form of multi-organ failure and death. Table 1 summarizes the number of operations.

**Table 1:** Demographic distribution surgical operation at King Abdullah University Hospital from 18/3/2020 to 18/4/2020.

Variables	Number	Percent (%)
	Mean ± SD	
**Sex**		
Male	62	33.9
Female	121	66.1
**Age (y)**	37.1 ± 20.7	
**Type of operation**		
Elective mandatory	102	55.7
Emergency	81	44.3
**Specialty**		
General Surgery	19	10.4
Obstetrics and Gynecology	76	41.5
Neurosurgery	16	8.7
Thoracic surgery	2	1.1
Otolaryngology	2	1.1
Urology	18	9.8
Pediatrics Surgery	7	3.8
Vascular Surgery	16	8.7
Maxillo-facial Surgery	3	1.6
Orthopedics	16	8.7
Ophthalmology	7	3.8
Cardiac surgery	1	0.5
**Trauma**	13	7.1
**Intraoperative complications**	1	0.5
**Postoperative complications**	7	3.8
**Surgical patients with COVID-19**	2	1.1
**Type of anesthesia**		
General anesthesia	105	57.4
Spinal anesthesia	63	34.4
Local anesthesia	15	8.2

In contrast and during the month preceding the lockdown, a total of 1622 operations were carried out. Among them, 1417 operations were elective and 205 was emergency. There is a huge difference in the number of operations in all specialty. This is summarized in Fig. 1.

**Fig. 1:**
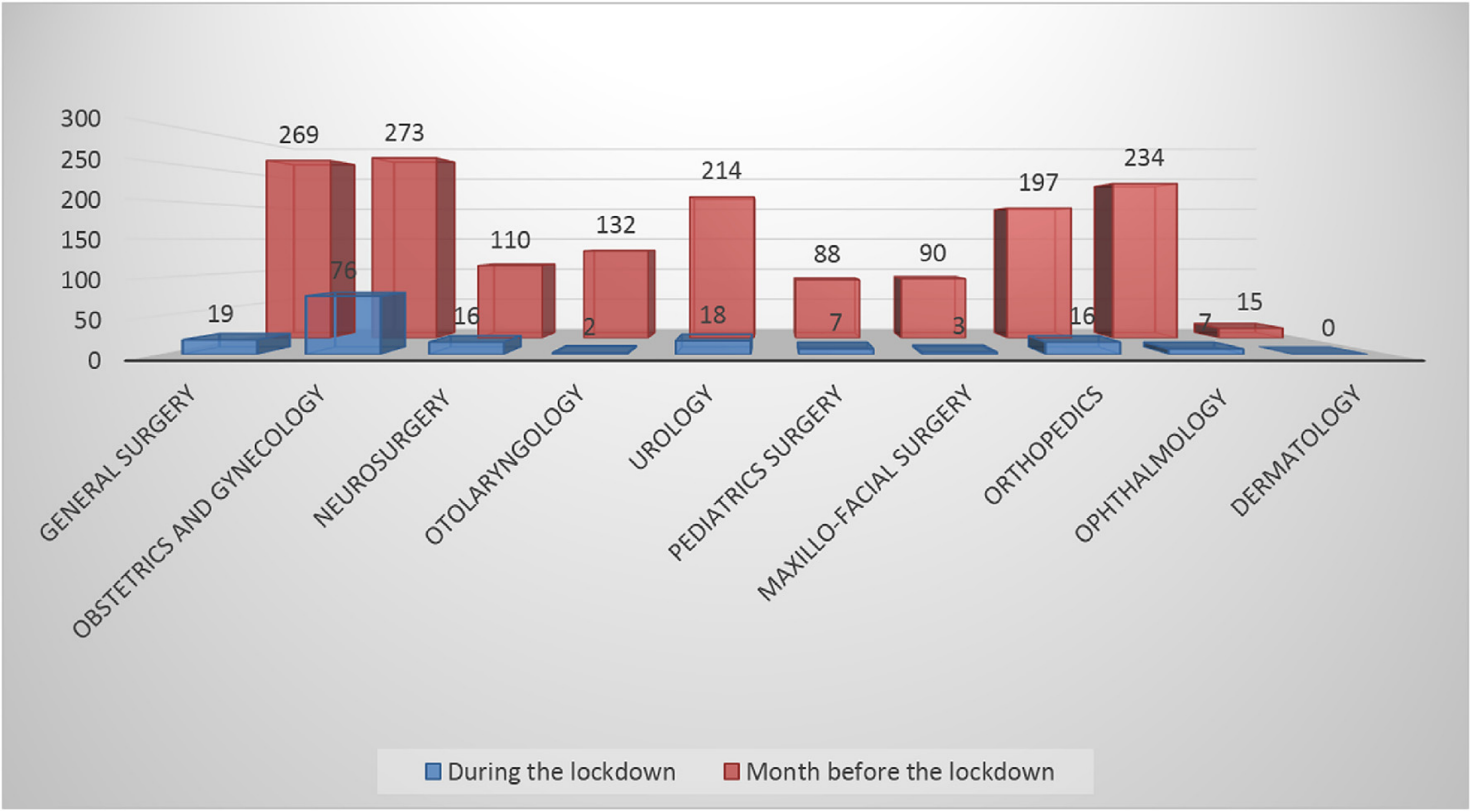
Chart compares the number of operations for many specialties.

Great efforts were done to postpone any operation that can be delayed. Nevertheless, in the near future, this situation could lead to a large amount of operation and pressure on the surgical services. In addition, this situation may develop many complicated cases in the near future due to the postpone of the operation.

## Provenance and peer review

Not commissioned, externally peer reviewed.

## Funding

No fund was received.

## Ethical approval

Not required.

## Consent

Not applicabe

## Author contribution

All authors contributed significantly and in agreement with the content of the article. All authors presented substantial contributions to the article and participated of correction and final approval of the version to be submitted.

## Registration of research studies

Thank you so much.

This is a letter to editor that describes the surgical practice in Jordan during COVID-19 pandemic.

## Guarantor

Dr Tagleb Mazahreh.

## Declaration of competing interest

The authors declare that they have no competing interests.

## References

[1] Rait J.S., Balakumar C., Montauban P., Zarsadias P., Iqbal S., Shah A. (2020). COVID-19 and surgery: running on good will or guilt?. Ann Med Surg (Lond)..

[2] Karampelias V., Spanidis Y., Kehagias I. (2020). Surgical practice and operative surgical strategies during the COVID-19 pandemic: a commentary [published online ahead of print, 2020 May 16]. Ann Med Surg (Lond).

[3] Feeley I., McAleese T., Clesham K. (2020). Foot and ankle service adaptation in response to COVID-19 and beyond [published online ahead of print, 2020 Apr 28]. Ann Med Surg (Lond).

[4] Bani Hani D., Alsharaydeh I., Bataineh A. (2020). Successful anesthetic management in cesarean section for pregnant woman with COVID-19. Am J Case Rep.

[5] Rana R.E., Ather M.H. (2020). Change in surgical practice amidst COVID 19; example from a tertiary care centre in Pakistan. Ann Med Surg (Lond)..

[6] Meraghni N., Benkaidali R., Derradji M., Kara Z. (2020). Orthopedic healthcare in the time of COVID-19: experience of the orthopedic surgery department at Mustapha Bacha Hospital, Algeria. Ann Med Surg (Lond).

[7] Patriti A., Eugeni E., Guerra F. (2020). What Happened to Surgical Emergencies in the Era of COVID-19 Outbreak? Considerations of Surgeons Working in an Italian COVID-19 Red Zone.

